# Improving protein domain classification for third-generation sequencing reads using deep learning

**DOI:** 10.1186/s12864-021-07468-7

**Published:** 2021-04-09

**Authors:** Nan Du, Jiayu Shang, Yanni Sun

**Affiliations:** 1 Computer Science and Engineering, Michigan State University, East Lansing, 48824 USA; 2 Electrical Engineering, City University of Hong Kong, Hong Kong, People’s Republic of China

## Abstract

**Background:**

With the development of third-generation sequencing (TGS) technologies, people are able to obtain DNA sequences with lengths from 10s to 100s of kb. These long reads allow protein domain annotation without assembly, thus can produce important insights into the biological functions of the underlying data. However, the high error rate in TGS data raises a new challenge to established domain analysis pipelines. The state-of-the-art methods are not optimized for noisy reads and have shown unsatisfactory accuracy of domain classification in TGS data. New computational methods are still needed to improve the performance of domain prediction in long noisy reads.

**Results:**

In this work, we introduce ProDOMA, a deep learning model that conducts domain classification for TGS reads. It uses deep neural networks with 3-frame translation encoding to learn conserved features from partially correct translations. In addition, we formulate our problem as an open-set problem and thus our model can reject reads not containing the targeted domains. In the experiments on simulated long reads of protein coding sequences and real TGS reads from the human genome, our model outperforms HMMER and DeepFam on protein domain classification.

**Conclusions:**

In summary, ProDOMA is a useful end-to-end protein domain analysis tool for long noisy reads without relying on error correction.

**Supplementary Information:**

The online version contains supplementary material available at (10.1186/s12864-021-07468-7).

## Introduction

Third-generation sequencing (TGS) technologies, such as Pacific Biosciences single-molecule real-time sequencing (PacBio) and Oxford Nanopore sequencing (Nanopore), produce longer reads than next generation sequencing (NGS) technologies. With increased read length, long reads can contain complete genes or protein domains, making gene-centric functional analysis for high throughput sequencing data more applicable [1–3]. In gene-centric analysis, often there are specific sets of genes in pathways that are of special interest, for example G protein-coupled receptor (GPCR) genes in intracellular signaling pathways for environmental sensing, while other genes in the assemblies provide little insight to the specific questions.

One basic step in gene-centric analysis is to assign sequences into different functional categories, such as families of protein domains (or domains for short), which are independent folding and functional units in a majority of annotated protein sequences. There are a number of tools available for protein domain annotation. They can be roughly divided into two groups depending on how they utilize the available protein domain sequences. One group of methods rely on alignments against the references. HMMER is the state-of-the-art profile search tool based on profile hidden Markov models (pHMM) [4, 5]. But the speed of the pHMM homology search suffers from the increase in the number of families. Extensive research has been conducted to improve the efficiency of the profile homology search [6].

The other group of tools are alignment-free [7]. Recent developments in deep learning have led to alignment-free approaches with automatic feature extraction [8–11]. A review of some available methods and their applications can be found in [12]. Of the learning-based tools, the most relevant one to protein domain annotation is DeepFam [9], which used convolutional neural networks (CNN) to classify protein sequences into protein/domain families. The authors showed that it outperformed HMMER and previous alignment-free methods on protein domain classification. Also, DeepFam is fast and the speed is not affected much by the number of families. For example, DeepFam is at least ten times faster than HMMER when 1,000 query sequences are searched against thousands of protein families [9]. Thus deep learning-based methods have advantages for applications that do not need detailed alignments.

Despite the success of existing protein domain annotation tools, they are not ideal choices for domain identification in error-prone reads. Although the sequencing accuracy of TGS platforms has improved dramatically, TGS data have lower per read accuracy than short-read sequencing [13]. The newest circular consensus sequencing (CCS) reads by PacBio Sequel II can reach high accuracy [14]. However, these reads exhibit a bias for indels in homopolymers [14]. In particular, there is still much room to improve for reads produced via direct RNA sequencing [13].

Insertion or deletion errors, which are not rare in TGS data, can cause frameshifts during translation [15]. Without knowing the errors and their positions, the frameshifts can lead to only short or non-significant alignments [16]. As the translation of each reading frame is partially correct, it also leads to poor classification performance for existing learning-based models. Our experimental results in “Experiments and results” section clearly showed this.

### Domain classification with error correction

Because sequencing errors remain an issue for TGS data, there are active developments of error correction tools for long reads [15, 17]. An alternative pipeline is therefore to apply tools such as HMMER and DeepFam to error-corrected sequences. Error correction tools can be generally divided into hybrid and standalone depending on whether they need short reads for error correction. Recently, several groups conducted comprehensive review and comparison of existing error correction tools [15, 17]. None of these tools can achieve optimal performance across all tested data sets.

Based on the recent reviews and also our own experimental results, there are two major limitations of applying error correction before protein domain classification. First, the performance of standalone tools is profoundly affected by the coverage of the aligned sequences against the chosen backbone sequences. When the coverage is low (e.g. the depth of sequencing <50X for LoRMA [18]), fewer regions of the reads can be corrected. Second, we found that error correction tools have difficulty correcting mixed reads from homologous genes within the same family, such as those from GPCR. The similarities between different genes/domain sequences can confuse the error correction method. For example, when we applied LoRMA to all the simulated GPCR reads, it failed to output any corrected sequence. Thus, in our experiments, we run the error correction tools for the sequences of each family separately in order to maximize their error correction performance, which is not practical in real applications. Details of these experiments can be found in “Can we rely on error correction?” section. In summary, error correction tools have unsatisfactory performance for data with low sequencing depth and data containing a mixture of homologous genes.

### Overview of our work

In this work, we designed and implemented ProDOMA, a deep learning based method to predict the protein domains from third-generation sequencing reads. By training a CNN-based model using 3-frame translation encoding of error-containing reads, ProDOMA is able to classify TGS reads into their correct domains with significantly better accuracy than existing domain classification tools. The main reason behind the improved performance for error-prone reads is that the deep learning model trained with a large number of simulated long reads is able to learn short but error-free motifs from different reading frames. The sequence logos of the most frequently activated filters from the three reading frames show that they share short and well-conserved motifs. In addition, we tested our model on remote homologues to examine whether this model is memorizing rather than learning the sequence patterns. The results showed that ProDOMA is superior to other models for remote homology search too.

Compared to previous works, ProDOMA has two merits in its usage. First, it does not require error correction. As a result, it has robust performance for low coverage data. Second, unlike previous deep learning works that were designed for classification, ProDOMA can also be used for detection by distinguishing targeted domain homologues from irrelevant sequences. The detection performance is better than HMMER after ProDOMA adopts a modified loss function from targeted image detection.

The classification accuracy of ProDOMA consistently outperformed the state-of-the-art method like HMMER and DeepFam across various error rates (from 1% to 15%) and its performance is not affected by the changed error rates and also the sequencing coverage. We tested it on real third-generation sequencing datasets, focusing on its function on detecting targeted domains using Outlier Exposure [19]. ProDOMA achieved higher recall with comparable precision to HMMER.

## Methods

**Fig. 1:**
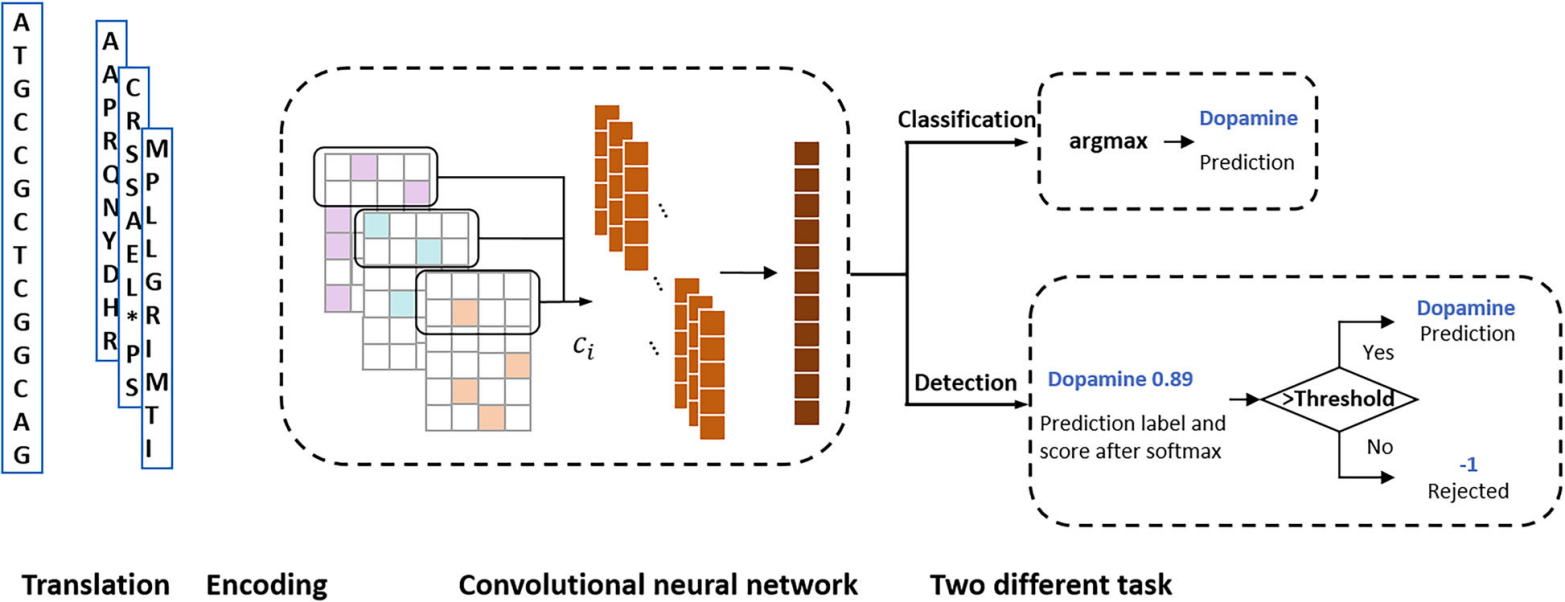
The overview of ProDOMA. The input sequence was translated and encoded to a 3-channel tensor. *c*_*i*_ is defined in Equation (1). In the classification task, the model directly outputs the family with the largest score as the prediction. In the detection task, the maximum of softmax score needs to be compared with a specified threshold to determine whether the input contains a trained domain family or should be rejected

Figure 1 sketches the architecture of ProDOMA. We chose the CNN because the convolutional filters can represent motifs, which are important for sequence classification [9]. There are many hyperparameters we can experiment in the network architecture. The empirical results show that the training data and the encoding methods can affect the performance significantly. Using error-containing sequences and 3-frame encoding leads to high classification accuracy for TGS reads. To exclude the unrelated coding or non-coding DNA sequences, we trained the CNN using a modified loss function so that out-of-distribution samples tend to have uniform distribution on softmax values.

### Encoding

With frequent insertion and deletion errors, the correct translation of a read is composed of fragments of different reading frames. In order to train the CNN to learn the conserved motifs from erroneous translations, we implemented and compared multiple encoding methods (see “Comparison of encoding methods and model structures” section). The empirical results show that the 3-frame encoding scheme achieved the best performance. In this scheme, each DNA sequence is translated into 3 protein sequences using 3 reading frames. To accommodate domain identification on the reverse strand, the reverse complement of the sequence is also used as input. Thus, ProDOMA considers 6 reading frames for each read. All the residues in the translated sequence are one-hot encoded using a 21-dimensional vector following IUPAC amino acid code notation. Then we combine three matrices into a single 3-channel tensor like an RGB image.

Given a translated sequence of length *n*, the encoded input is a tensor with size 3×*n*×21. The pseudo-code of 3-frame encoding can be found in Algorithm 1.


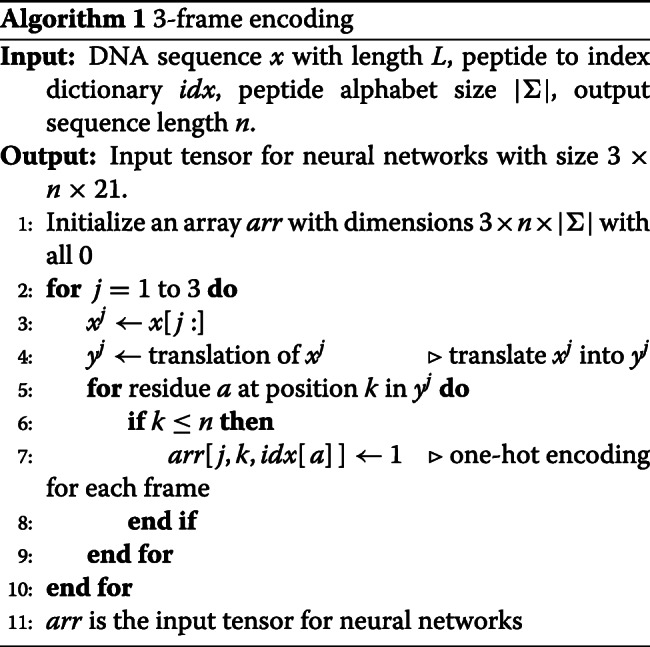


### Convolutional neural networks

ProDOMA consists of two convolutional layers, one max-over-time pooling layer, one hidden linear layer, and one linear output layer with the softmax function. For a multi-channel input that we have from 3-frame encoding, we transform the output *arr* from Algorithm 1 into a feature value using the following equation.

1 $$ c_{i} = f\left(\sum_{j=1}^{3} \mathbf{w}_{j}\cdot \mathbf{arr}[j][i:i+h-1][1:|\Sigma|] + b \right)  $$

*b* is the bias term and *h* is the filter size. *f* is the activation function ReLU [20]. The filter consists of three 2D matrices **w**_*j*_ for *j*=1,2, and 3, corresponding to three reading frames. **arr**[*j*][*i*:*i*+*h*−1][1:|*Σ*|] defines a 2D window of size *h*×|*Σ*| for the one-hot matrix with reading frame *j*. We applied filters repeatedly to each possible window of the input one-hot matrix to produce the feature map. Then the max-over-time pooling is applied to the feature map to capture the maximum value max(*c*_*i*_) as the feature from this particular filter. The max-over-time pooling is flexible with different input length.

Equation 1 described how a single filter in the convolutional layer works. In our application, we used multiple filters with various sizes to extract features of different lengths.

ProDOMA has two convolutional layers. The first convolutional layer uses consistent filter size to extract low-level motif-like patterns directly from the 3-frame encoding input. Then the second convolutional layer extracts high-level, intricate patterns with varying distance from the output of the first convolutional layer. By repeatedly applying the operations, we can finally generate a feature map. Then the max-over-time pooling was applied to keep the most important features. Dropout [21] is also used after pooling to prevent overfitting and to learn robust features. A two-layer classifier with softmax function transfers the features to a vector of probabilities over each label. For classification, we select the label with the maximum probability as the prediction from ProDOMA.

### Comparison of encoding methods and model structures

We also tested other encoding methods with similar model structure to ProDOMA: (1) DNA one-hot encoding, which directly transfers DNA sequence to a one hot encoding matrix of size *L*×4. For a fair comparison, we used filter sizes that are 3 times as long as we used for 3-frame encoding; (2) 3-branch model, where we constructed a network architecture with three branches processing each of the 3-frame translated protein sequence separately. Each of the branches consists of identical convolution layers, and all the parameters are shared between the same layer of 3 branches. In other words, Eq. (1) becomes *c*_*i*_(*j*)=*f*(**w**_*j*_·**arr**[*j*][*i*:*i*+*h*−1][1:|*Σ*|]+*b*) for *j*=1 to 3. In the 3-branch model, each branch models the translated protein sequences separately before the merging layer right before the two-layer classifier. In contrast, in our 3-frame encoding, all three translated protein sequences were processed and combined by the 3-channel convolution filter in the first convolutional layer.

Our experimental results show that 3-frame encoding is a better encoding scheme, possibly because it can effectively encode the original DNA sequence information and also helps convolutional filters extract useful features for prediction of the protein domains (See results in “Performance with different architectures” section). In addition, our experiments show that changing the order of the input reading frames does not affect the classification accuracy.

### Detecting out-of-distribution inputs

We have described how ProDOMA predicts the domain labels for given DNA reads using CNN and softmax. However, with the close-set property of softmax, the classifier will always assign a label for the input sample, even if the input is not related to any label in the model (we call such inputs out-of-distribution samples, compared to in-distribution samples). For example, in RNA-Seq data, not every read encodes targeted domain families in the model. In real applications, this close-set property will lead to an undesired high false-positive rate. To address the problem, we adopt Outlier Exposure (OE) [19] with a threshold on softmax prediction probability [19] to distinguish the out-of-distribution inputs from in-distribution ones.

#### The threshold baseline

Usually, the samples from the out-of-distribution dataset tend to have small softmax values because their normalized probabilities are more uniformly distributed than the samples from the in-distribution dataset.

**Fig. 2:**
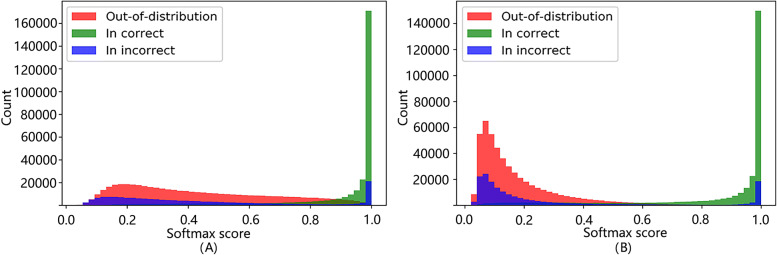
The histograms of maximum softmax values for in-distribution and out-of-distribution samples from base model (**a**) and model with Outlier Exposure (**b**). “In correct”: in-distribution samples with correct classification. “In Incorrect”: in-distribution samples with incorrect classification

Following [19], we extracted the maximum value of the softmax probability vector from the output of ProDOMA for each input sample. We separated the in-distribution samples from the out-of-distribution samples by specifying a threshold on the maximum softmax probability. A holdout dataset with both in-distribution and out-of-distribution samples was used to empirically determine the best threshold that can produce the largest *F*_1_ score: $F_{1} = 2 \cdot \frac {\text {precision}\cdot \text {recall}}{\text {recall}+\text {precision}}$. Then this learned softmax threshold is used to reject any sample with smaller softmax values. The performance of this baseline model is shown in Fig. 2a.

#### Outlier exposure

To further improve the performance of the out-of-distribution sample detection, we adopt the Outlier Exposure (OE) method introduced by [19]. As we discussed previously, we expect the out-of-distribution samples to have uniformly distributed softmax probabilities for all classes. However, as such inputs were never processed in training, sometimes the model will yield unexpected high confidence prediction for out-of-distribution inputs (Fig. 2a). To address the problem, we expose the model to out-of-distribution samples in the training process to let the model effectively learn the heuristics for detecting out-of-distribution inputs. Compared with the threshold baseline, we need to introduce a new dataset with out-of-distribution samples in the training process.

Given a model *g* and the learning objective $\mathcal {L}$, the objective of OE is to minimize the original loss function with an auxiliary loss term to regularize the out-of-distribution examples. OE can be formulated as minimizing the following objective [19]:

2 $$ \mathbb{E}_{(x,y)\sim \mathcal{D}_{\text{in}}}\left[\mathcal{L}(g(x),y)+\lambda\mathbb{E}_{x'\sim \mathcal{D}_{\text{out}}}\left[\mathcal{L}_{\text{OE}}(g(x'),g(x),y)\right]\right]  $$

3 $$ \mathcal{L}_{\text{OE}} = -\sum_{i}\mathcal{P}(i)\ln \frac{\mathcal{Q}(i)}{\mathcal{P}(i)}  $$

$\mathcal {D}_{\text {in}}$ is the original in-distribution dataset, $\mathcal {D}_{\text {out}}$ is the out-of-distribution dataset for OE. In the original classification task, we use the cross-entropy loss function $\mathcal {L}$. In order to force the out-of-distribution samples to have uniform distribution on all labels, we minimize the KL-divergence between out-of-distribution and the uniform distribution as shown in Eq. (3). $\mathcal {Q}$(i) is the predicted distribution of out-of-distribution samples from the model and $\mathcal {P}$(i) is a normalized uniform distribution. In the experiment, we use *λ*=0.5 for the coefficient of the auxiliary loss. More detailed and comprehensive description of OE can be found in the original publication [19].

Figure 2 presents the distribution of the maximum softmax score for each input sequence with and without OE for the threshold calibration dataset we used in “Human genome dataset” section. Without OE, there are still a lot of out-of-distribution samples with large softmax scores (0.5 to 1). With OE, most of the out-of-distribution samples accumulate with small softmax scores (0 to 0.4). With OE, the overlapping area between the two distributions (red vs combined green and blue) is decreased from 26.06% to 21.99%. In addition, for the in-distribution samples with small softmax values, their classification results tend to be wrong (blue in Fig. 2). Thus, using OE can provide better classification accuracy at a cost of rejecting some in-distribution samples.

## Experiments and results

To evaluate ProDOMA, we applied ProDOMA on both simulated and real datasets: a simulated PacBio G protein-coupled receptor (GPCR) coding sequences (CDS) dataset [7], and two real third-generation sequencing datasets of the human genome [22, 23]. GPCR is a large protein family that is involved in many critical physiological processes, such as visual sense, gustatory sense, sense of smell, regulation of immune system activity, and so on [24]. In addition, GPCR is a very diverse set of protein sequences and thus can pose challenges for classification. It consists of 8,222 protein sequences belonging to 5 families, 38 subfamilies, and 86 sub-subfamilies. Following DeepFam, all the experiments are conducted on the sub-subfamilies.

We compared the performance of ProDOMA with HMMER and DeepFam, which are representatives of alignment-based and alignment-free domain classification tools. In both experiments, ProDOMA was trained with simulated PacBio reads from the GPCR CDS downloaded from NCBI. The simulation was conducted using a popular simulation tool PBSIM [25] with default setup and error rates from 1% to 15%. Following their instructions and design principle, HMMER and DeepFam were trained using the correct protein sequences in the GPCR dataset.

In our first experiment, we tested ProDOMA and its alternative implementations on simulated PacBio reads. In the second experiment, we tested ProDOMA on real PacBio and Nanopore reads from human data. All specific commands, parameters, and output of our experiments can be found along with the source code of ProDOMA.

### Experiments on simulated PacBio GPCR CDS dataset

The reference coding sequences of each sub-subfamily are divided into 80% training samples and 20% test samples. The number of reference sequences in each class is shown in Table S1 in Supplementary File 1. Then we used PBSIM to generate simulated PacBio reads with 80X coverage on the plus strand for training and test samples with specified error rates. As a result, the training set has 939,888 simulated reads, and the test dataset has 228,388 simulated reads for 86 sub-subfamilies, respectively. Our strategy of conducting simulation after splitting the coding sequences can guarantee that there is no overlap of the GPCR CDS sequences between the training and test datasets, which is important for meaningful evaluations. In our experiments, we used 5-fold cross validation. Thus, the above training and testing dataset construction process was repeated five times.

#### Performance with different architectures

**Fig. 3:**
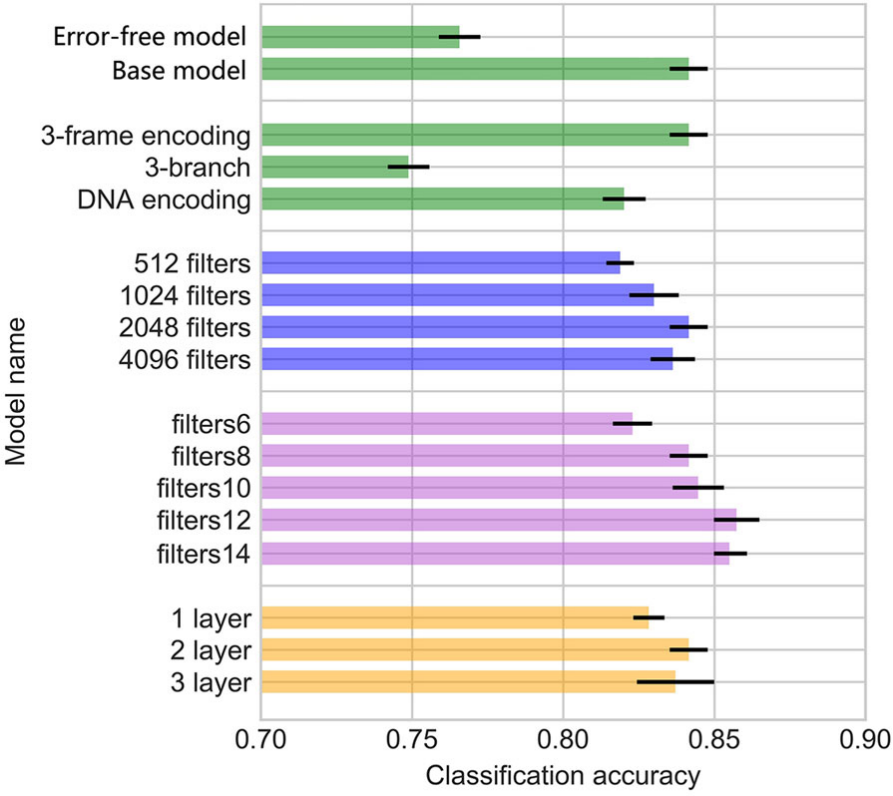
The mean and standard deviation of classification accuracy of different network architectures. Different colors represent different group of comparison: **green** bars for encoding and dataset; **blue** bars for number of filters; **purple** bars for different filter sizes; **orange** bars for different convolutional layers. **Error-free model**: the training data only contain the error-free reads; **Base model**: the training data contain both error-free reads and reads with error rate of 10%; **3-frame encoding**: the encoding strategy in the base model; **3-branch**: 3 branches structure for translated reads; **DNA encoding**: use one-hot encoding of DNA reads as input; **512 filters**: use 512 filters in total in the 2nd convolutional layer; **1024 filters**: use 1024 filters in total in the 2nd convolutional layer; **2048 filters**: use 2048 filters in total in the 2nd convolutional layer; **4096 filters**: use 4096 filters in total in the 2nd convolutional layer; **filters6**: filter sizes of 2nd convolutional layer =[6,9,12,15,18,21,24,27]; **filters8**: filter sizes of 2nd convolutional layer =[8,12,16,20,24,28,32,36]; **filters10**: filter sizes of 2nd convolutional layer =[10,15,20,25,30,35,40,45]; **filters12**: filter sizes of 2nd convolutional layer =[12,18,24,30,36,42,48,54]; **filters14**: filter sizes of 2nd convolutional layer =[12,18,24,30,36,42,48,54,60]; **1 layer**: only keep the last convolutional layer; **2 layer**: use two convolutional layers; **3 layer**: add an extra convolutional layer with 64 filters of size 3

We conducted a series of experiments by varying the key components in our base models: the training data, the number of convolutional layers, the number of convolution filters, the size of convolution filters, and different encoding strategies. Totally, we compared 14 different combinations of hyperparameters or architectures and two different types of training data in the experiments. Except for the error-free model, all these experiments were trained and tested on reads with an error rate of 10%. The error-free model was trained on error-free reads and tested on reads with an error rate of 10%. We listed all variations and their accuracy on the testing set in Fig. 3. The highest accuracy is 86.74%, which is achieved by using 3-frame encoding with two convolution layers. Based on the comparison, the key factors affecting the performance are the encoding strategies, the size of filters, and the type of training data.

**Comparisons of the types of training data** Using error-containing reads as training data led to higher accuracy than the error-free model. Using reads with errors in training data has the same function as the data augmentation, which is widely adopted in computer vision [26]. Because the TGS data usually contain sequencing errors, the data augmentation method will help the model prevent over-fitting and be more robust to the error.

**Comparisons of filter configurations** Using 2 layers achieved higher accuracy than 1 layer. The extra layer helps the neural network extract more complex patterns such as interactions of the lower level features. However, the “deeper” model with more layers is more difficult to optimize. That is the possible reason why the average accuracy of the 3-layer model is lower than the base model (with 2 layers), but the highest accuracy achieved is higher than our base model.We found that the additional convolutional filters increased the performance for protein domain prediction. The improvement is saturated when we have more than 2,048 filters.Increasing the size of filters can also help improve the performance of the models. With larger filter sizes, the neural network can capture long-range features at a cost of training time. The result suggests the importance of choosing the right filter size, which is not explored in previous works [9, 27].

#### The input order of the Reading frames does not change the classification accuracy

**Fig. 4:**
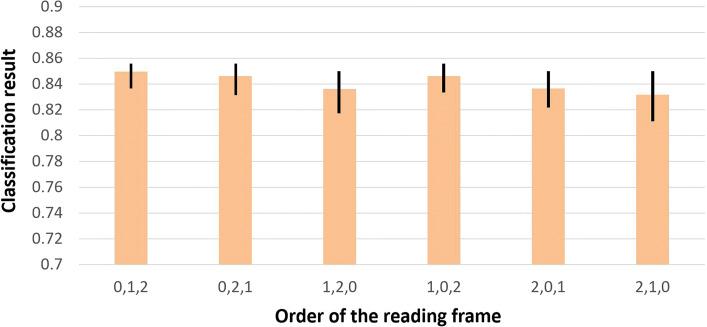
The mean, min, and max value of classification accuracy using different orders of reading frames as input. X-axis: order of reading frames as input. Y-axis: classification accuracy

Reads starting from different positions in the same transcript can have different reading frames corresponding to the same translation. In our model training process, the three channels always take translations of reading frame 0, 1, and 2 of a read as input. It is thus fair to ask whether this specific order affects the classification performance. We investigate this question by inputting different orders of reading frames of test sequences to our trained model. Thus, we generated 6 inputs from each reads with different frame orders. As a result, 1,370,328 validation samples are tested in the experiment. Figure 4 shows the classification accuracy of 5-fold cross validation using different reading frame orders as input.

**Fig. 5:**
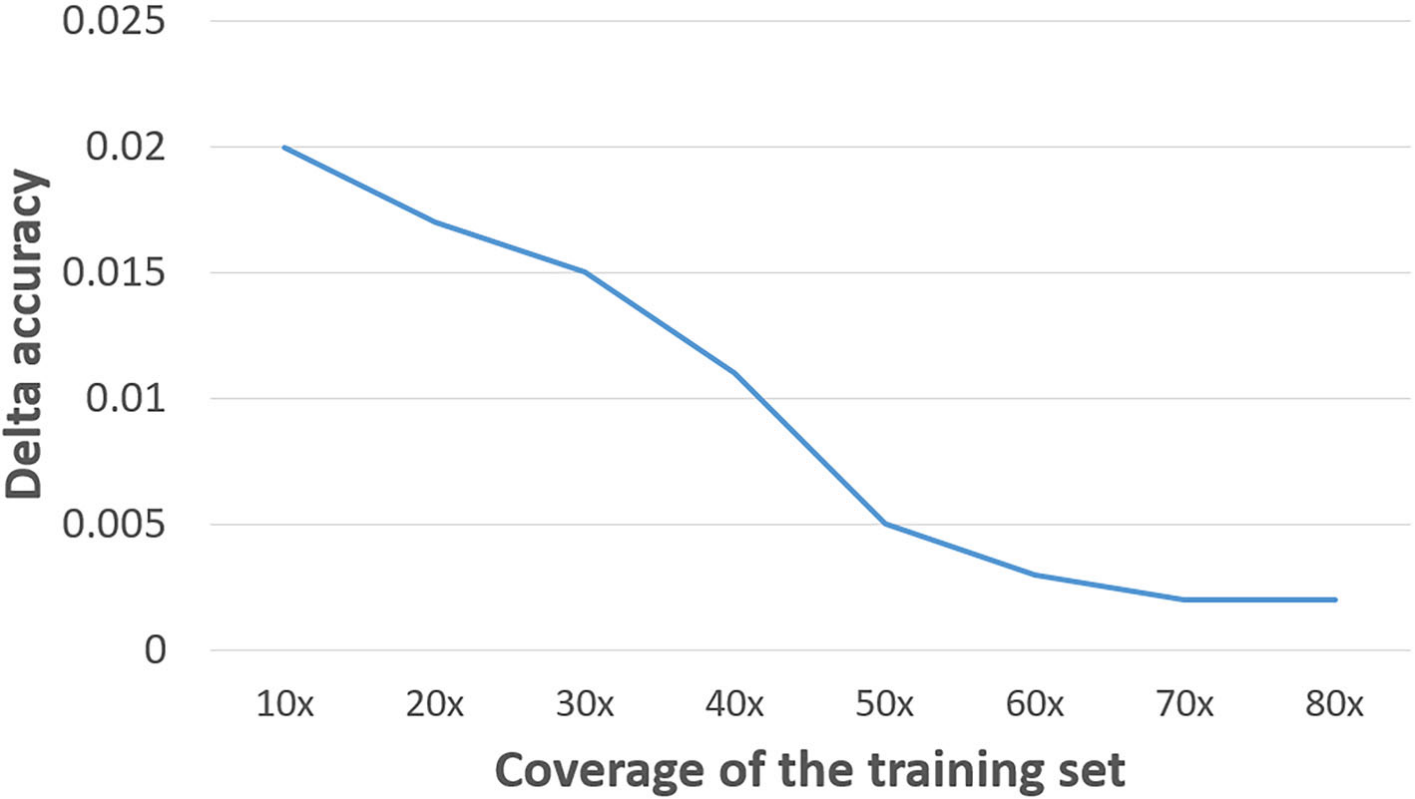
The accuracy range of different reading frame orders vs. the coverage of the training set. X-axis: coverage of the training set. Y-axis: Delta accuracy (the difference of the highest and lowest accuracy between different reading frame orders)

The classification accuracy using different reading frames is generally consistent. Figure 5 shows that with the increase of training set coverage (from 10x to 80x), the difference of the highest and lowest accuracy between the 6 orders decreases. the lowest difference of accuracy is 0.001 (80x training set).

#### Comparison with HMMER and DeepFam

There are many other existing tools like Selective top-down [28], RPS-BLAST[29] and UProC [30] for protein domain classification. For alignment-free tools, we choose DeepFam [9] because of its superior performance. And as shown in [9, 28], DeepFam achieves the best performance in alignment-free models. Also, HMMER [4, 5] is one of the most widely used alignment-based methods for protein domain classification and has been proven to be reliable on different datasets. The experiment results shown in [30] also shows that it achieves better performance on longer reads. Both HMMER and DeepFam are also well maintained and can be easily applied to conduct experiments using different types of reads. Thus, we choose HMMER and DeepFam as the benchmark tools.

Following the design principles and the instructions of HMMER and DeepFam, the training of HMMER and DeepFam was conducted using correct protein sequences, rather than DNA sequences. The test sequences are simulated long reads from reference CDs. Their three-frame translations are used as input to HMMER and DeepFam. As long as one of the three translated sequences is classified to the correct sub-subfamily, we call this a correct prediction.

The classification accuracy of ProDOMA and DeepFam was measured using 5-fold cross-validation. As it is tedious to perform 5-fold cross validation for HMMER, we used all 5-fold correctly translated protein sequences to train the pHMM model, which will favor HMMER as the trained model has seen the test sequences. MAFFT [31] was used to generate the multiple sequence alignment for each sub-subfamily. Then we used hmmbuild in the HMMER package to build pHMM models for each sub-subfamily. For each test DNA sequence, 3-frame translations were applied to get three peptide sequences. All the translated sequences were tested using hmmscan against all 86 pHMM models we built.

**Fig. 6:**
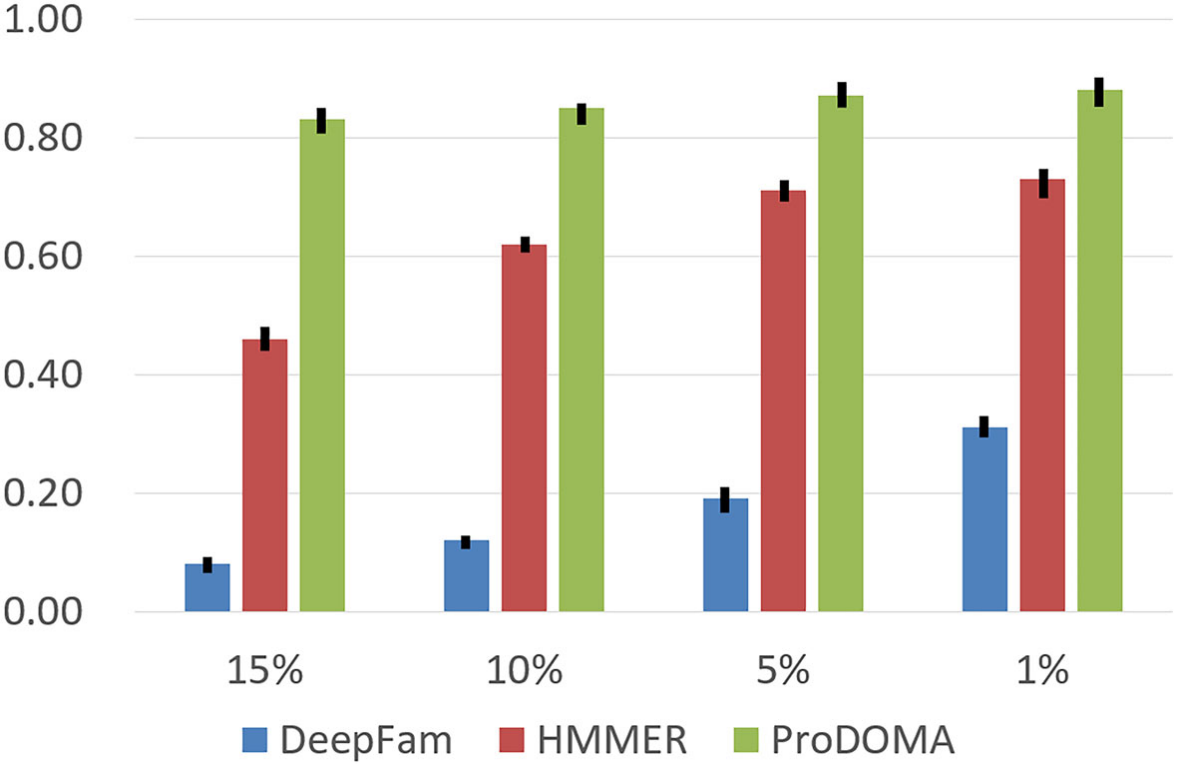
The mean, min, and max value of classification results of ProDOMA, HMMER, and DeepFam on classifying four sets of simulated long reads with different error rates. Each test set contains roughly 228K simulated reads from 86 sub-subfamilies. X-axis: different error rates. Y-axis: classification accuracy

Figure 6 compares the classification performance of all methods on the simulated PacBio reads. For this data set, our method achieved better performance for datasets with different error rates. The high error rates heavily impacted the performance of HMMER and DeepFam. It is expected because the profile HMM search is much more sensitive to frameshifts caused by gaps, and DeepFam is designed for classifying relatively complete error-free protein sequences.

#### Can we rely on error correction?

As there are error correction tools for long reads [17], existing domain classification tools such as HMMER can be applied on error-corrected reads. We conducted an experiment to test whether family classification using corrected reads can achieve comparable performance to classification of error-free sequences.

Hybrid error correction tools tend to perform better than self-correction tools. However, not every sequence project has budget and manpower to generate both short read and long read libraries and data. Based on these recent reviews [15, 17], we chose LoRMA [18], a more recently published self-error correction tool, with the lowest error rate after correction [17]. Note that LoRMA failed to generate outputs if we use all the simulated reads as input, probably because of the similarity between the homologous GPCR sequences or the large graph produced by the reads. Thus, we run LoRMA for each set of reads simulated from the same reference sequence of GPCR in order to achieve the best error correction performance. Similar to other groups’ observations, LoRMA discarded a large number of reads during error correction when the coverage is not high, which will jeopardize abundance estimation. As shown in Table 1, although the number of reads remained after error correction increased along with the increase of the coverage, it still discarded a large number of reads (e.g. 75.9% reads are discarded when the coverage is 30x). Also, the error correction tools became significantly slower with the increase of the coverage.

**Table 1:** The number of reads before and after error correction and the running time of LoRMA

**Coverage**	Before	After	Percentage	Time (h:m:s)
10x	88124	7897	8.9%	14:28:07
20x	161504	30884	19.1%	26:24:10
30x	235083	56677	24.1%	39:12:02

It becomes very slow with the increase of the coverage

We recorded the domain classification results for corrected reads in Table 2. HMMER achieved the best accuracy for corrected reads. ProDOMA achieved comparable accuracy on corrected reads. With or without error correction, ProDOMA’s performance is consistent with the change of the coverage. Without relying on error correction, ProDOMA can conduct domain classification for all reads and thus lead to a more accurate estimation of the domain/family abundance. As DeepFam will assign a family to each translation, it is unknown which one should be chosen in practical applications. We regard a read as a correct classification as long as one of the translation is correctly classified.

**Table 2:** The classification accuracy comparison between ProDOMA, HMMER and DeepFam on corrected reads

	All simulated reads			Corrected reads		
	ProDOMA	HMMER	DeepFam	ProDOM	HMMER	DeepFam
10x	87.12%	62.12%	25.06%	95.05%	97.42%	69.06%
20x	86.47%	63.25%	25.01%	94.94%	97.59%	77.36%
30x	86.69%	62.56%	25.18%	94.79%	98.16%	80.76%

The first column is the coverage

#### Performance of remote homology search

**Fig. 7:**
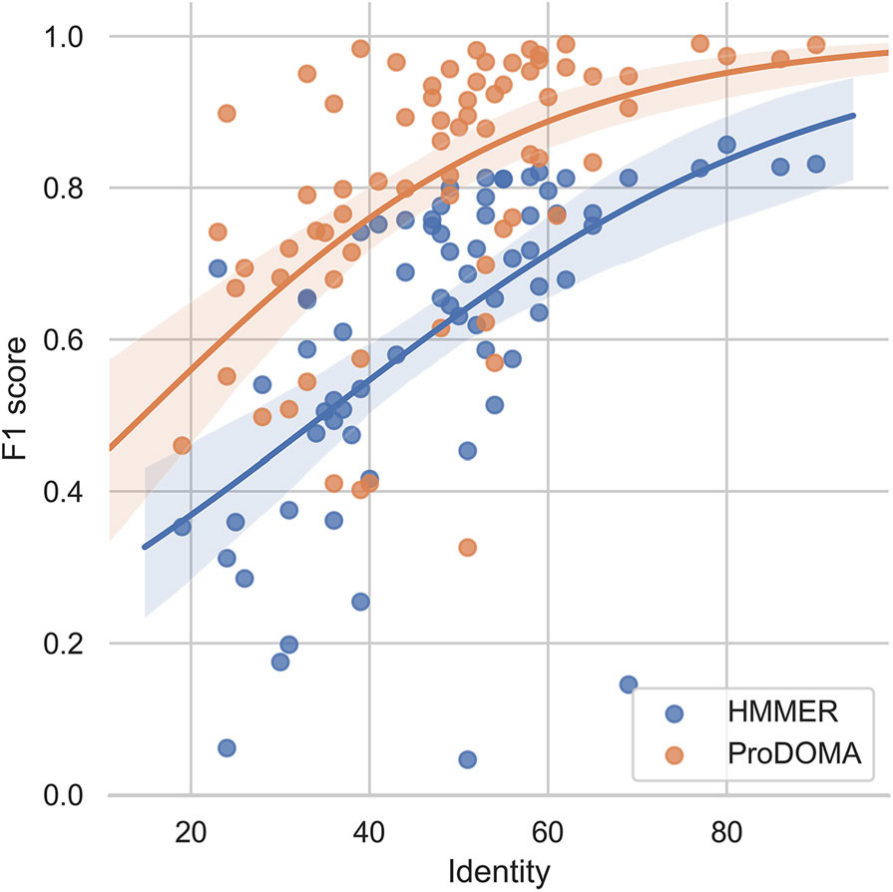
Scatter plot and their logistic regression curves for all sub-subfamilies. The error bar (regions surrounding the curves) is the 95% confidence interval of the fitting. Y-axis: F1 score of the test sequences. X-axis: intra-class identity

As GPCR is a large protein family, some of their coding sequences can show high diversity, posing a challenge for both alignment-based and alignment-free homology search. We plot the change of F1 score of each protein family with the change of the intra-class identity in Fig. 7. As DeepFam’s performance is inferior to others, we did not include DeepFam in this experiment. The intra-class identities were calculated using the alistat tool provided in the HMMER package. The sub-subfamilies with low identities are more likely to have remote homologues. F1 score is the harmonic mean of recall and precision. For one protein family A, let its true member sequences be set *A*^+^ and the predicted sequence set be *A*^*pred*^. The recall is thus $ \frac {|{A^{+}}\cap {A^{pred}}|}{|A^{+}|}$. Precision is $ \frac {|{A^{+}}\cap {A^{pred}}|}{|A^{pred}|}$.

Figure 7 shows that both HMMER and ProDOMA suffer from low intra-class identity but the performance of ProDOMA deceases slower with the decrease of the identity. One possible reason is that deep learning can learn more degenerate features that are hard to model by HMMs.

#### Comparison of the running time

With a large amount of data generated by third-generation sequencing platforms, we require both high accuracy and efficiency for the algorithms. We run ProDOMA, DeepFam, and HMMER using Intel^®^ Xeon^®^ Gold 6148 CPU with 20 cores at the High-Performance Computing Center at Michigan State University. We also tested ProDOMA and DeepFam with NVIDIA^®^ Tesla^®^ V100 GPU with Apex acceleration library (HMMER doesn’t support GPU). For each method, We measured its execution time by averaging 5 independent trials with randomly selected 10,000 sequences.

As shown in Table 3, in CPU, HMMER with the default setup runs much faster than ProDOMA. One reason is that with high sequencing error rates, the alignment against many candidate sub-subfamilies cannot pass the filter stage of HMMER, skipping the expensive pHMM alignment. By turning off all filters, the sensitivity of HMMER increases, but at a large cost in speed. With –max (turning off all filters), HMMER is much slower than deep learning-based methods. With GPU acceleration, the running time of ProDOMA is much shorter than the running time of HMMER with the default setup.

**Table 3:** The average elapsed time to predict sub-subfamily labels of 10,000 simulated PacBio reads for each method

Setup	ProDOMA	DeepFam	HMMER	HMMER −max
CPU	1168.78s	276.74s	312.13s	3470.04s
GPU	25.71s	20.37s	unavailable	unavailable

### Human genome dataset

To evaluate ProDOMA’s performance on real third-generation sequencing dataset, we tested ProDOMA on the *H. sapiens* 10x Coverage data from PacBio [23] and Oxford Nanopore Human Reference Datasets Rel6 [32]. In this experiment, as the real genome sequencing data contains non-GPCR coding sequences, we will also test the performance of ProDOMA on detecting out-of-distribution samples.

**Training dataset** The training dataset is the same as the previous experiments: long reads simulated using PBSIM. To apply Outlier Exposure, we constructed a dataset that mixed the previous 5-fold training dataset with an outlier dataset. In order to generate the outlier dataset with similar distributions to the real out-of-distribution samples, we simulated a PacBio human genome dataset from GRCh37/hg19 human reference genome [33]. Then we kept the simulated reads that cannot be aligned to any GPCR CDS by BLASR in the outlier dataset [34]. As a result, the outlier dataset has 800,000 simulated reads. Then we retrained ProDOMA with OE as discussed in Methods.

**Test dataset** We used two test datasets: a PacBio RS II test dataset from PacBio SMRT Sequencing for CHM1TERT human cell line; and a Nanopore test dataset from Oxford Nanopore MinION on CEPH1463 (NA12878/GM12878, Ceph/Utah pedigree).

We determine the ground truth of these test reads using sequence similarity, which favors alignment-based tools. We first aligned all reads against GPCR CDS dataset using BLASR. We extracted the reads with alignment length longer than 60% of aligned CDS sequence as our in-distribution test samples. For each sample, the ground truth is given by the label of aligned CDS.

We also randomly selected reads that were not aligned to any GPCR CDS as our out-of-distribution samples. To test different portions of non-GPCR reads, we generated datasets consisting of 1%, 5%, 10%, and 50% GPCR reads. For reads longer than 3,000 bps in the test dataset, we cropped each read into fragments that are at most 3,000 bps to be consistent with training dataset.

#### Out-of-distribution test using PacBio and Nanopore reads

As the purpose of this experiment is to evaluate the performance of GPCR CDS detection, we computed the recall, precision, and F1 score of each tool on labeling reads from the 86 classes. Recall is the ratio of correctly predicted reads to the total number of reads from the 86 classes. Precision is the ratio of the reads from the 86 classes to the total number of reads predicted with the 86 class labels. F1 score is the harmonic mean of recall and precision and we reported micro-F1 score for our multi-classification model.

We benchmarked the OE model with HMMER, which is highly accurate in distinguishing protein domains from other sequences. As shown in Table 4, both methods have low recall but high specificity because many reads are rejected and not classified into any of the 86 classes. Table 4 also shows that although there is some slight difference in the performance when the proportion of GPCR changes, the overall performance is consistent. This is because our model is robust in removing OOD reads in general.

**Table 4:** The performance of protein domain prediction with out-of-distribution examples using ProDOMA with Outlier Exposure (OE), and HMMER on the real PacBio and Nanopore dataset

Proportion	Method	PacBio			Nanopore		
		Recall	Precision	F1-score	Recall	Precision	F1-score
1% GPCR	HMMER	0.1403	0.9574	0.2446	0.3902	**0.9876**	0.5593
	ProDOMA	**0.4581**	**0.9872**	**0.6258**	**0.4727**	0.9796	**0.6376**
5% GPCR	HMMER	0.1537	0.9411	0.2642	0.4015	0.9754	0.5513
	ProDOMA	**0.4712**	**0.9921**	**0.6389**	**0.4615**	**0.9872**	**0.6289**
10% GPCR	HMMER	0.1491	0.9513	0.2577	0.3988	**0.9917**	0.5688
	ProDOMA	**0.4473**	**0.9842**	**0.6150**	**0.4886**	0.9843	**0.6529**
50% GPCR	HMMER	0.1494	0.9507	0.2583	0.3984	**0.9825**	0.5670
	ProDOMA	**0.4479**	**0.9837**	**0.6154**	**0.4836**	0.9731	**0.6458**

Still, ProDOMA with OE achieved significant improvement on recall while the precision is comparable with HMMER (Table 4). In general, both methods have better performance on the Nanopore dataset. Note that in the whole pipeline, we only used simulated PacBio reads for training. This result suggests that our strategy is robust with different types of long reads.

## Discussion

In this work, we showed that ProDOMA can render better accuracy for domain classification in long noisy reads. The major differences between the architecture of ProDOMA and other CNN-based sequence classification models are the coding strategy designed for erroneous reads and the modified loss function to reject out-of-distribution samples. In order to interpret why this 3-frame encoding works, we further investigate the feature extracted by convolutional filters.

Following the methods adopted by the previous research [9, 35], we visualized the convolution units activated for each family. We used the model that was trained in previous experiments and fed it with the test sequences belonging to this family. Then we collected all the sequence fragments that activated the convolution units. We extracted the results from the most frequently activated convolution units and used Weblogo [36] to generate the logos from these sequences. Since we translated the original input DNA sequences using three reading frames, we have 3 logos associated with the 3 frames.

**Fig. 8:**
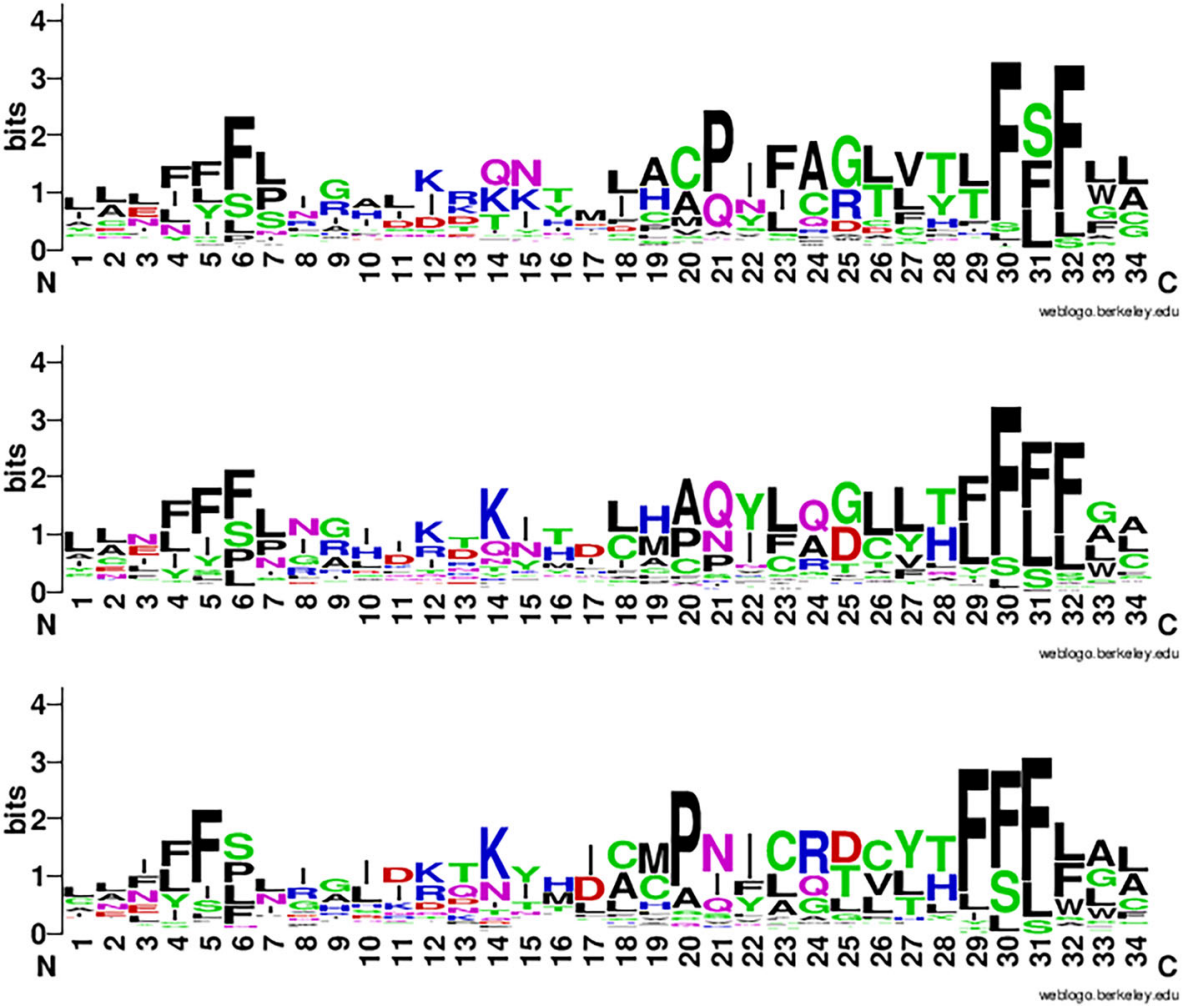
Logos derived from most frequently activated convolutional filters from the three reading frames for Latrophilin

**Fig. 9:**
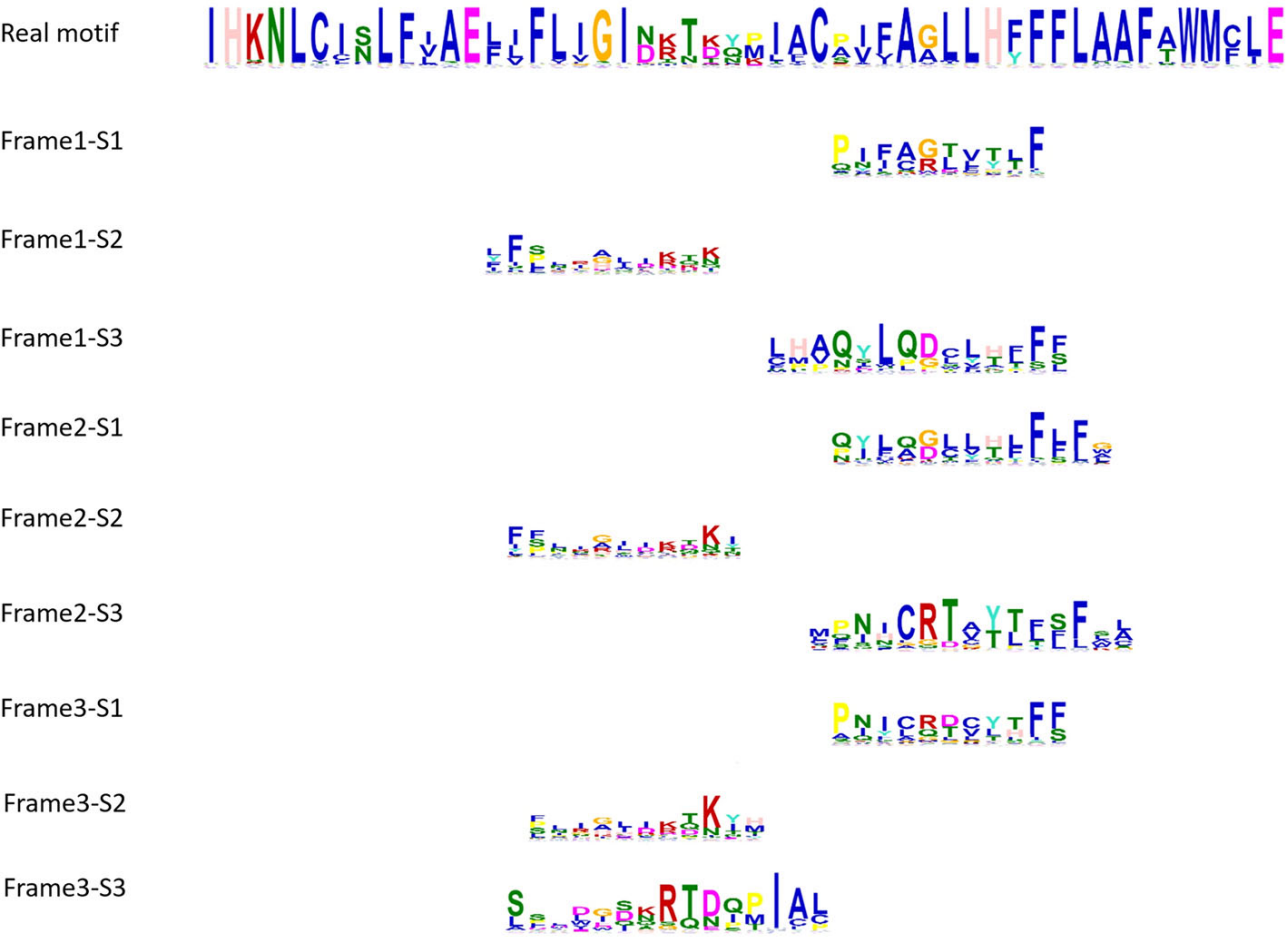
Motif analysis for Latrophilin. Real motif: the motif derived from the error-free homologous sequences in Latrophilin. FrameX-SX: sub-motifs derived from most frequently activated convolutional filters in different frames (their logos are shown in Fig. 8. These sub-motifs are aligned to the real motif based on the motif similarities returned by MEME

We showed the three logos of one class named Latrophilin in Fig. 8. They don’t share high global similarity because they are translated using different reading frames. But they have sub-motifs with high similarities with the real motif from the original member sequences of Latrophilin. These sub-motifs are aligned to the real motif in Fig. 9. These figures indicate that the filters from different channels learned short but conserved motifs from the underlying families. More examples of the full-length filters can be found in Figure S2 to Figure S4 in Supplementary File 1.

As the model is trained using sequences with 3,000 nt, padding is applied to all inputs shorter than 3,000. Sequences longer than 3,000 will be cut into substrings of length 3,000, which will then be fed into the model. Thus our CNN model cannot accurately detect the start and end positions of domains. Instead, a model that can accurately assign weights for each base is needed for detecting the entering and existing positions of each domain. We plan to explore deep learning-based annotation our future work.

In summary, ProDOMA provides a complementary tool to current third-generation sequence analysis pipelines on gene-centric function analysis. It can directly identify protein domains in long noisy reads without relying on error correction and its performance is robust to low coverage data and can tolerate higher error rates than other domain classification tools.

## Supplementary Information

**Additional file 1** Supplementary File 1

## Data Availability

The datasets, source code and the trained model used during the current study are available in the ProDOMA repository: https://github.com/strideradu/ProDOMA.

## Abbreviations

TGS: Third-generation sequencing
PacBio: Pacific Biosciences single-molecule real-time sequencing
Nanopore: Oxford Nanopore sequencing
NGS: next generation sequencing
GPCR: G protein-coupled receptor
pHMM: profile hidden Markov models
CNN: Convolutional neural networks
CCS: circular consensus sequencing
CDS: coding sequences
OE: Outlier exposure
