# Supplementary material for: Improving protein domain classification for third-generation sequencing reads using deep learning
Source: BMC Genomics. 2021 Apr 9;22:251. doi: 10.1186/s12864-021-07468-7 (PMC8033682; doi:10.1186/s12864-021-07468-7)
Supplement: Supplementary file 1 — Additional file 1 Supplementary File 1 [file 12864_2021_7468_MOESM1_ESM.pdf]

SUPPLEMENTARY FILE 1

ProDOMA: improve PROtein DOMAin classification for third-generation sequencing reads using deep learning

Nan Du[1†], Jiayu Shang[2†] and Yanni Sun[2*]

*Correspondence: yannisun@cityu.edu.hk
[1]Computer Science and Engineering, Michigan State University, 48824 East Lansing, USA
Full list of author information is available at the end of the article
†Equal contributor

1 Details of the data sets

The dataset used in the experiment is shown in Table S1. The table is sorted by the number of reference sequences in each class. The minimum class BOSS and ExtraCale has only 2 sequences. The Maximum class Olfactory has more than 3,000 sequences in it. Our experimental results showed that the classification accuracy is generally lower for small classes (Figure. S1).

class name	No.	Class name	No.	Class name	No.	Class name	No.
BOSS	2	Methuselah	37	MelaninConc	69	Histamine	169
ExtraCalc	2	Glucagon	38	Oxytocin	71	Melaton	222
Adrenomedullin	3	cAMP	39	Vasopressin	72	Prostacyclin	181
Gonadotrophin	3	PutPher	39	Cholecystokinin	81	BLT2	182
CalcLike	6	Kiss1	40	Growth	82	Duffy	183
Secretin	7	Anaphylatoxin	43	Purinergic	84	Prolactin	186
Neuromedin	8	Orexin	47	Vasotocin	84	Bombesin	187
Proteinase	9	GABA	50	Thyro	97	Prostaglandin	285
AlphaFac	14	MuscAcetyl	65	Calcitonin	98	Melanocortin	228
SubstanceP	17	Latrophilin	53	Tachykinin	110	Adrenergic	248
Thyrotropin	19	NeuropeptideFF	53	Angiotensin	112	Chemokine	248
Melanocyte	21	PACAP	54	Interleukin8	116	Serotonin	333
SubstanceK	21	EMR1	55	Cannabinoid	122	Taste	345
Thrombin	25	Allatostatin	56	FollicleStim	169	C5A	540
UrotensinII	29	Galanin	75	Adrenocorticotropic	141	Traceamine	557
GrowthHorm	31	Vasoactive	59	GRHR	145	Bradykinin	601
Prokineticin	32	LysoEdg2	63	Octopamine	147	GlutaMeta	975
Cadherin	25	Parathyroid	63	Adenosine	194	Adrenoreceptor	1395
Neurotensin	34	Platelet	67	Neuropeptide	156	Olfactory	3012

Table S1: The dataset used in our experiment. No. is number of reference sequences in the corresponding class.

2 Implementations and hyperparameters

ProDOMA was implemented by Python3 using PyTorch [1] with Apex [2] acceleration. The model was trained using a gradient-descent optimization algorithm with adaptive estimates of moments called Adam [3]. The training target is to minimize multiple class cross-entropy loss function. We use five-fold cross validation to select the best model. In the splitting-5-folds program, we randomly split each class into five folds and then construct the train-set and test-set rather than randomly splitting the whole dataset into 5-folds.

We also implemented several commonly used techniques for optimizing deep neural networks, such as mini-batch gradient descent, He initialization [4], and hyperparameter tuning. There are several hyperparameters including the number and the

size of convolutional filters, the number of input and output features in the linear layer, dropout rate, learning rate, and batch size. We tried our best to search for the optimal parameters based on the training data. The default hyperparameters were set as follows: batch size = 256, learning rate = 0.001, dropout rate = 0.5. For the first convolutional layer, the size of the filters is 3, and the number of filters is 64. For the second convolutional layer, the size of the filters is $8, 12, 16, 20, 24, 28, 32, 36$ with 256 filters of each size. Also, for the hidden linear layer, the number of output features is 512. Given the data size we used, we found our model converges around 1 to 2 epochs (˜5,000 to 10,000 steps) training, so we trained all models two epochs with validating every 102,400 samples (400 steps) to select the best model.

3 Performance of each class

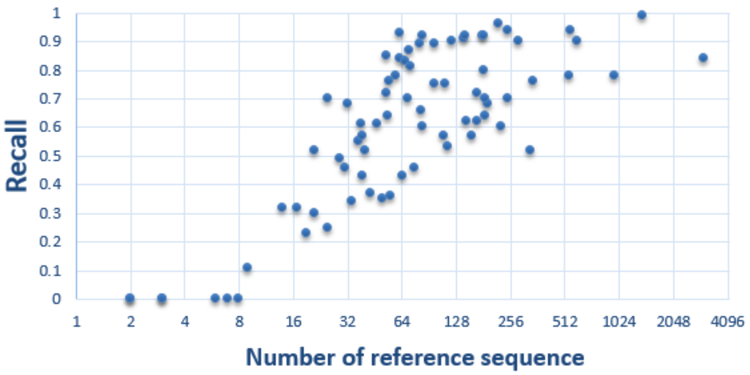

Figure S1: The relationship between performance and the number of reference sequences in the class. X-axis: The number of reference sequences in the class. Y-axis: The recall of the corresponding class

Figure S1 shows the relationship between performance and the number of reference sequences in the class. In the experiment, we will use 80% sequences to train the model and the remained 20% will be used as a validation set. To receive the performance of each class, we only use samples in one class at a time and run ProDOMA to achieve the result. Thus, we use metric recall, which is calculated by $\mathcal{TP}/(\mathcal{TP}+\mathcal{FN})$, to measure the performance.

As shown in Figure S1, with the number of reference sequences increases, The recall for the corresponding class becomes higher. This is because deep learning is a data-hungry algorithm. The model can learn better if we have more high-quality training samples. The number of sequences in each class is shown in Table S1.

4 Convolution filters

From the logos, we can found that the convolutional filters did learn some conserved regions of protein families. However, the logo is very noisy compared to the results reported in previous studies. When the input has insertions and deletions, a well-conserved region may appear in all three frames due to frameshifts, so the filter of all three channels sometimes may need to extract similar patterns. That is why some of the 3-frame logos show similar patterns across different frames.

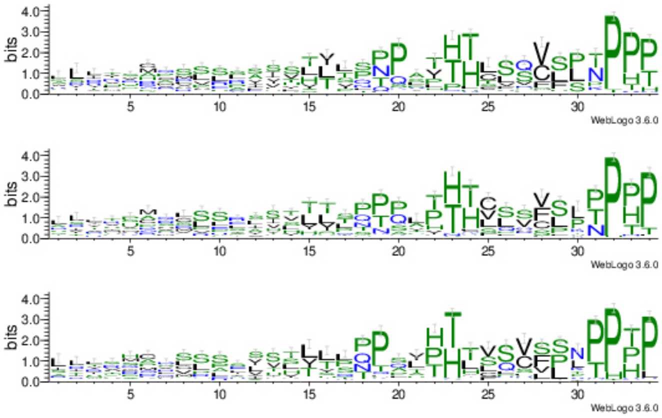

Figure S2: Most activated convolutional filters (index: 1689) for Platelet.

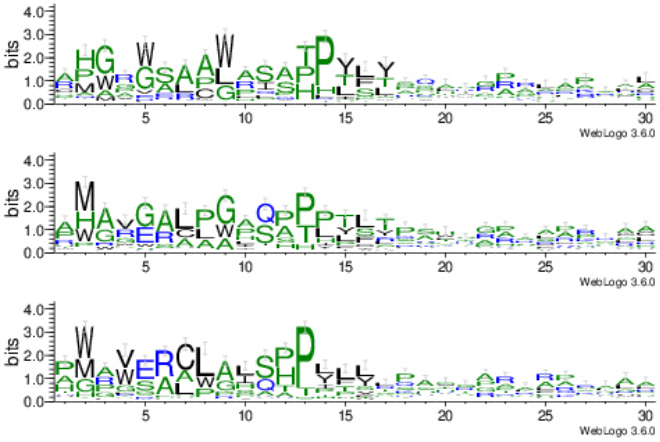

Figure S3: Most activated convolutional filters (index: 1382) for Prostacyclin.

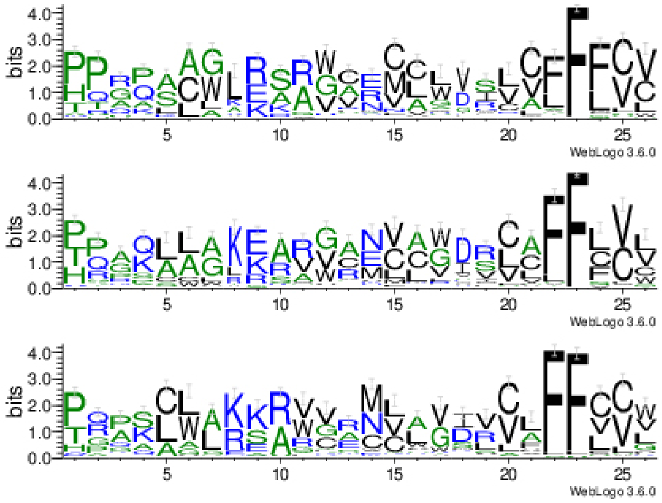

Figure S4: Logos derived from most frequently activated convolutional filters (index: 1033) for Cholecystokinin.

Author details
[1]Computer Science and Engineering, Michigan State University, 48824 East Lansing, USA. [2]Electronic Engineering, City University of Hong Kong, Hong Kong, China SAR.

References

1. Paszke, A., Gross, S., Chintala, S., Chanan, G., Yang, E., DeVito, Z., Lin, Z., Desmaison, A., Antiga, L., Lerer, A.: Automatic Differentiation in PyTorch. In: NIPS Autodiff Workshop (2017)
2. NVIDIA Corporation: Apex (2019). https://github.com/NVIDIA/apex
3. Kingma, D.P., Ba, J.: Adam: A method for stochastic optimization. arXiv preprint arXiv:1412.6980 (2014)
4. He, K., Zhang, X., Ren, S., Sun, J.: Delving deep into rectifiers: Surpassing human-level performance on imagenet classification. In: Proceedings of the IEEE International Conference on Computer Vision, pp. 1026–1034 (2015)
